# Enhanced anti-tumor activity of transferrin/folate dual-targeting magnetic nanoparticles using chemo-thermo therapy on retinoblastoma cancer cells Y79

**DOI:** 10.1038/s41598-023-49171-5

**Published:** 2023-12-15

**Authors:** Elaheh Sadri, Sepideh Khoee, Samaneh Moayeri, Bahareh Haji Ali, Vahid Pirhajati Mahabadi, Sakine Shirvalilou, Samideh Khoei

**Affiliations:** 1 Finetech in Medicine Research Center, Department of Medical Physics, School of Medicine, Iran University of Medical Sciences, P.O. Box: 1449614525, Tehran, Iran; 2 Department of Medical Physics, School of Medicine, Iran University of Medical Sciences, Tehran, Iran; 3 Department of Polymer Chemistry, School of Chemistry, College of Science, University of Tehran, Tehran, Iran; 4 Cellular and Molecular Research Center, Iran University of Medical Sciences, Tehran, Iran; 5 Neuroscience Research Center, Iran University of Medical Sciences, Tehran, Iran

**Keywords:** Biochemistry, Biotechnology, Cancer, Cell biology

## Abstract

Malignant neoplasms are one of the main causes of death, especially in children, on a global scale, despite strenuous efforts made at advancing both diagnostic and therapeutic modalities. In this regard, a new nanocarrier Vincristine (VCR)-loaded Pluronic f127 polymer-coated magnetic nanoparticles conjugated with folic acid and transferrin (PMNP-VCR-FA-TF) were synthesized and characterized by various methods. The cytotoxicity of these nanoparticles was evaluated in vitro and ex vivo conditions. The in vitro anti-tumor effect of the nanoparticles was evaluated by colony formation assay (CFA) and reactive oxygen species (ROS) in Y79 cell line. The results showed that nanoparticles with two ligands conferred greater toxicity toward Y79 cancer cells than ARPE19 normal cells. Under an alternating magnetic field (AMF), these nanoparticles demonstrated a high specific absorption rate. The CFA and ROS results indicated that the AMF in combination with PMNP-VCR-FA-TF conferred the highest cytotoxicity toward Y79 cells compared with other groups (P < 0.05). PMNP-VCR-FA-TF could play an important role in converting externally applied radiofrequency energy into heat in cancer cells. The present study confirmed that dual targeting chemo-hyperthermia using PMNP-VCR-FA-TF was significantly more effective than hyperthermia or chemotherapy alone, providing a promising platform for precision drug delivery as an essential component in the chemotherapy of retinoblastoma.

## Introduction

As the most prevalent type of ophthalmologic cancer in children, retinoblastoma can result in death if left untreated^1^. The likelihood of survival is heavily dependent on timely detection and proper treatment^2^. However, the advent of intra-arterial and intraocular chemotherapy in recent years has significantly enhanced the treatment of retinoblastoma, raising the likelihood of survival^3^. Among the most successful chemotherapeutic drugs utilized to treat retinoblastoma is vincristine sulfate^4^. The inherent potential of Vincristine to combat tumors is due to its interaction with tubulin, resulting in disintegration of the microtubules of the mitotic apparatus, and the subsequent arrest of metaphase cell division^5^. Unlike non-specific treatments with conventional chemotherapy, local delivery of anticancer agents is beneficial to enhance local drug concentrations and minimize unwanted side effects of chemotherapy^6^. A critical shortcoming of chemotherapy is systemic toxicity. The dose-limiting toxic effect of vincristine is neurotoxicity, which may affect peripheral motor, sensory nerve, cranial motor, and autonomic nerves^7^. Local delivery of anticancer drugs to the tumor site may reduce the toxic effect of the drug on healthy tissues, and as a result, could minimize the severity of side effects^8^. To overcome this problem, nanoparticles of various materials such as biodegradable polymers have been adopted to improve intraocular drug delivery and storage, potentially improving treatment of retinoblastoma^9^. There are two fundamental strategies for utilizing nanoparticles (NPs) in targeted drug delivery, namely passive and active targeting^10^. Active targeting relies on the natural interaction between ligands on the NP surface and cellular targets, which has been found to be more effective in treating human cancers than passive targeting^11^. Biological ligands bind to specific receptors on the target cell surface, leading to increased internalization of drug-loaded NPs and improved therapeutic effectiveness^12^.

Such ligands, including Folic acid ligands, offer unlimited availability, low cost, and low immunogenicity without the risk of toxicity or immune response for clinical application^13^. Therefore, FA-targeted drug delivery to various cancer cell types can maximize therapeutic efficacy while minimizing side effects^14^. In contrast, transferrin (Tf) is a glycoprotein present in the blood that binds the transferrin receptor (TfR), facilitating the transportation of iron into cells through receptor-mediated endocytosis^15^. Malignant cells require more iron and therefore overexpress the transferrin receptor, with up to 100 times more expression than in normal cells^16^. Transferrin receptor is appealing for tumor targeting and it is also expected that the simultaneous application of two transferrin/folic acid ligands will have better efficacy for targeting cells. Regardless, the efficacy of nanoparticle delivery systems that utilize a single-ligand is limited due to the intricate nature and heterogeneity of the tumor microenvironment. Compared to single ligands, double ligands are more likely to be taken up by cells due to multivalent effects^17^. Employing two distinct ligands in the delivery system to target different receptors, whether in the same cell type or entirely different cells, could be a novel approach to improve targeted drug delivery to tumor cells. The objective of this method is to enhance the effectiveness of cytotoxic drugs in destroying cancer cells^18^. In our study, the folic acid ligand (FA) and transferrin ligand (Tf) are used. FA can target folic acid receptors (FRs) and Tf can also target transferrin receptors (TfRs), which are selectively overexpressed on the surface of cancer cells, in contrast to normal cells^19^. The combination of two targeting ligands can improve selectivity and increase VCR uptake of Y79 cancer cells. In addition, triblock copolymer micelles comprise a hydrophobic center that serves as an enclosure to contain hydrophobic agents such as drugs and a hydrophilic shell that provides water stability^20^. To achieve this goal, we employed the Pluronic F127 block copolymer, which is amphiphilic and composed of blocks of poly(ethylene oxide) (PEO) and poly(propylene oxide) (PPO) arranged in a PEO-PPO-PEO configuration, to deliver therapeutic concentrations of VCR to retinoblastoma cells. Hyperthermia has long been used as a therapeutic approach among other treatments for cancer. Past studies have indicated that tumor cells are more sensitive to heat than normal cells^21,22^. Applying temperatures above 43 °C to heat-sensitive tumor cells not only makes the cells respond better to chemotherapy, but also causes direct cytotoxic effects^23^. In recent times, magnetic nanoparticles have been utilized as nanoscopic heat sources for thermal targeting of tumor cells without harming normal tissues^24^. In magnetic hyperthermia, an alternating magnetic field is applied to magnetic nanoparticles, the magnetic momentum of the nanoparticles combines with the oscillating field, and the absorbed energy is converted to heat in the target tumor tissue. The thermogenic process of the nanoparticles comprises three stages: Neel recovery, which involves energy release due to the rotation of nanoparticles’ magnetic moment; Brownian recovery, which involves physical processes that release energy in nanoparticles; and heat loss due to hysteresis^20,25^.

Based on this premise, here, we synthesized VCR-loaded pluronic-coated iron oxide nanoparticles consisting of FA and TF as dual ligands (VCR-PMNP-FA-TF) for targeted delivery, sustained release and combinatorial chemo-hyperthermia therapy for cancer cells. To improve the targeting efficiency, double-ligand guided nanocarriers were proposed. Targeting of two receptors on the surface cell could lead to greater affinity and specificity for carriers. Biodegradable polymers can be loaded with vincristine as a chemotherapeutic agent to diminish adverse effects and systemic toxicity of chemotherapeutic agents. Magnetic nanoparticles in the form of nanoheater capable of delivering targeted heat to tumor cells have not yet been investigated for the treatment of retinoblastoma. Next, we investigated the therapeutic effect of magnetic chemotherapeutic hyperthermia using PMNP-VCR-FA-TF nanoparticles on the retinoblastoma cell line Y79. A soft agar colony assay was used to examine cell death through magnetic hyperthermia in Y79 cells. Next, we evaluated the generation of reactive oxygen species (ROS) following treatment. We compared how the Y79 Retinoblastoma cell line and the retinal pigment epithelial cells (ARPE-19 cell line) responded to the treatment.

## Results

### Synthesis and characterization of drug-loaded and drug-free PMNP-FA-TF nanoparticles

**Figure 1:**
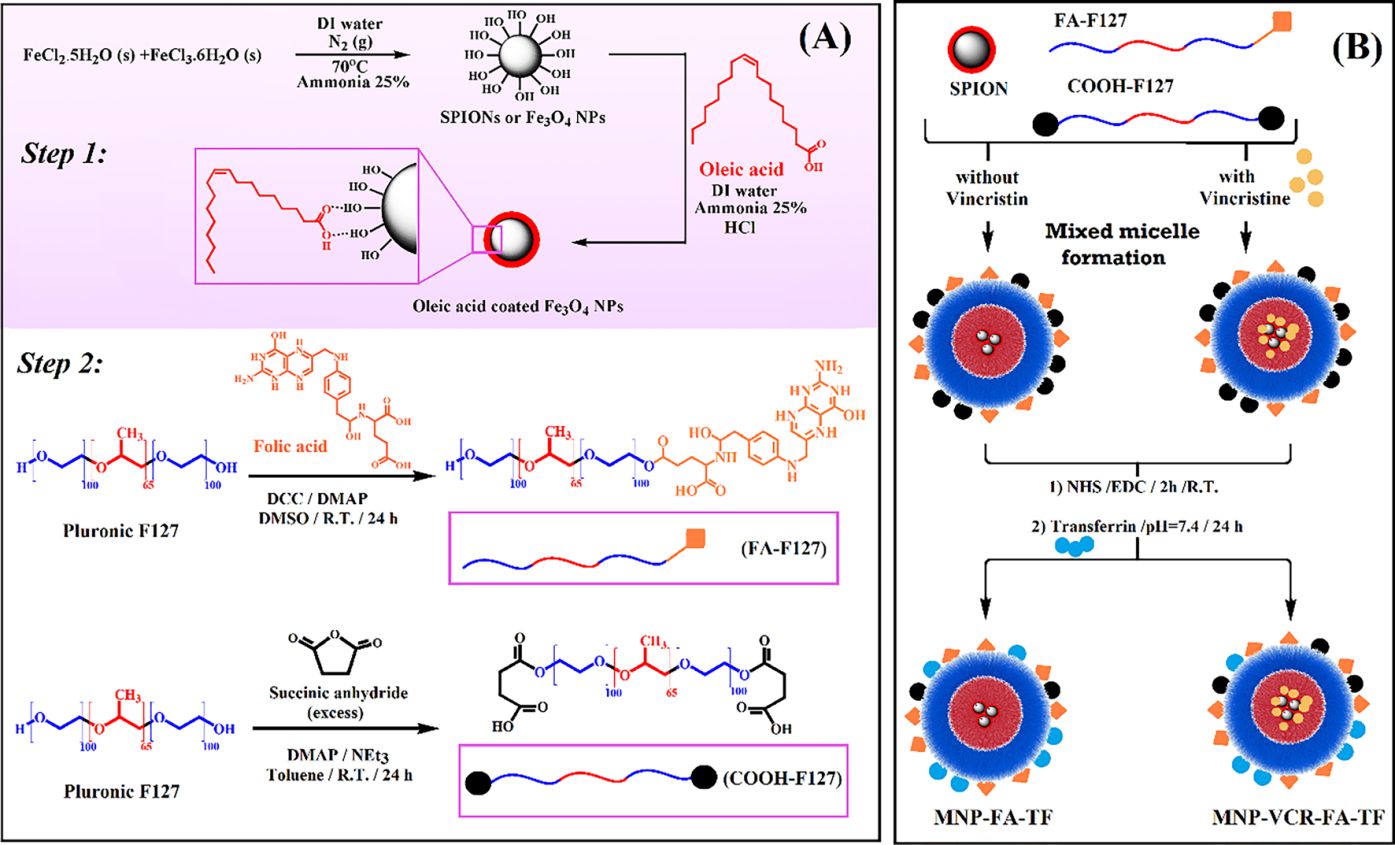
Synthetic steps of SPIONs, FA-F127, and COOH-F127 (**A**) and schematic illustration of final nanoparticles preparation (**B**).

Oleic acid (OA)-coated superparamagnetic iron oxide NPs were prepared via two steps: chloride salts of iron were used to synthesize the bare SPIONs in the presence of ammonia solution, and oleic acid was utilized as a commonly used surfactant for preparation of more stable dispersion of synthesized SPIONs (Fig. 1A, Step 1). Oleic acid provides monodisperse magnetite nanoparticles, stabilizes their dispersion, and prevents self-aggregation of them^26^. In the next step, folic acid-functionalized pluronic F127 and carboxylic acid terminated F127 were synthesized through the esterification method (Fig. 1A, Step 2). Mixed micelle formation occurred by nanoprecipitation of the carboxylic acid terminated F127 and folic acid functionalized pluronic F127 in the presence of a dispersion of SPIONs in water, according to Fig. 1B. Inserting the hydrophobic Vincristine in the above-mentioned process results in the formation of drug-loaded nanoparticles. To have a dual active targeting agent besides a passive one (SPIONs), Transferrin as a receptor was subjected to reaction with acid groups existing on the surface of drug-free and drug-loaded nanoparticles.

**Figure 2:**
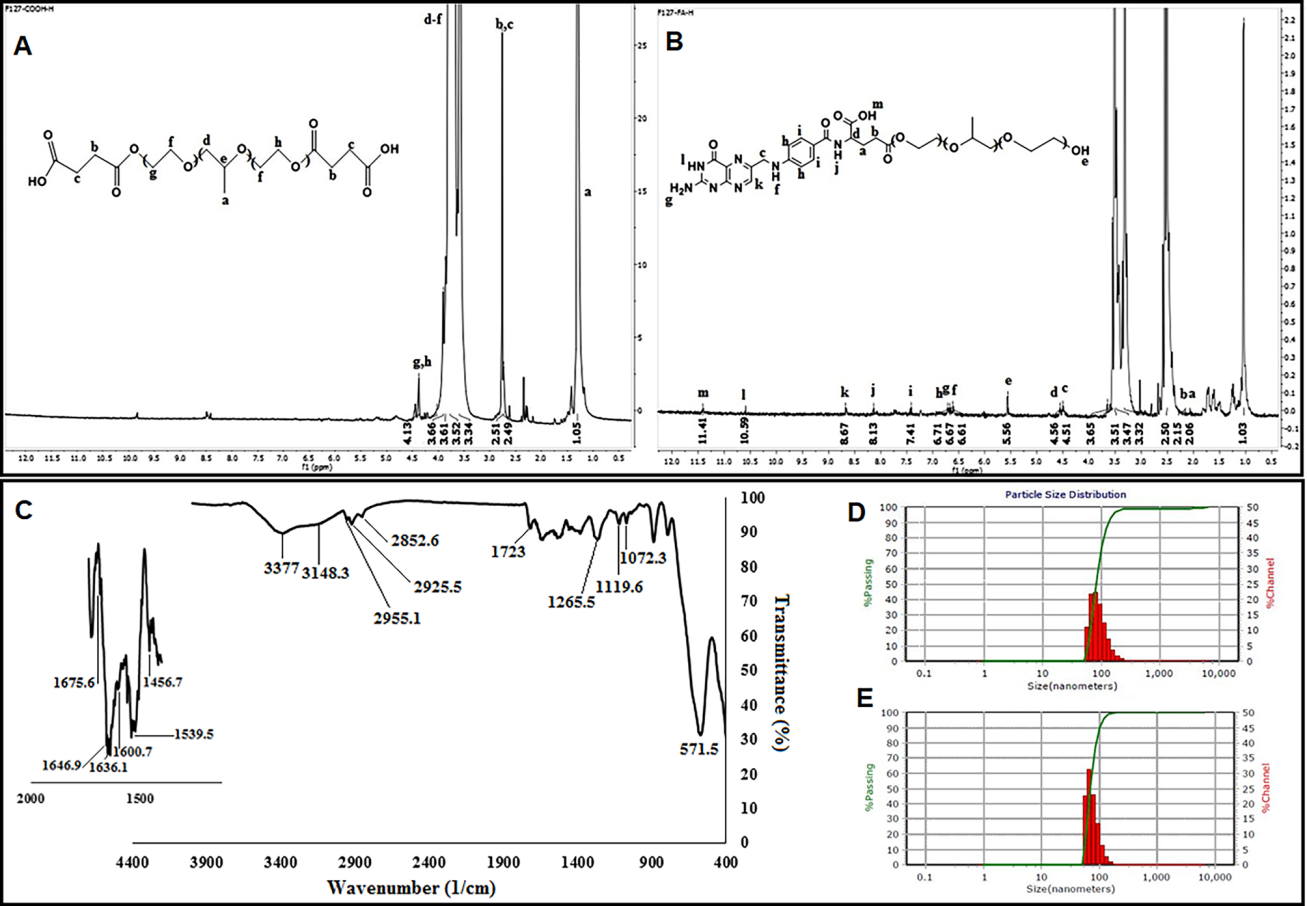
The 1HNMR spectra of (**A**) carboxyl functionalized Pluronic F127 polymer and (**B**) FA-conjugated Pluronic F-127 polymer in CDCl_3,_ FT-IR spectrum of Transferrin conjugated nanoparticles (PMNP-FA-TF) (**C**), nanoparticle size distribution of PMNP-FA-TF (**D**), PMNP-VCR-FA-TF (**E**).

Hydrogen-1 nuclear magnetic resonance (1HNMR) spectrum of COOH-F127 (Fig. 2A) illustrated a peak at 1.05 ppm related to the methyl groups of pluronic acid (a). Multiple resonances at 3.34–3.66 ppm (d–f) and the characteristic peaks at 4.13 ppm (g, h) are attributable to the C–H bonds of pluronic acid. The resonance of succinic anhydride CH_2_ groups that appeared at 2.49 (b) and 2.51 (c) ppm confirmed the carboxylic acid functionalization of pluronic acid F127 polymer. Figure 2B depicts the 1HNMR spectrum of the folic acid functionalized pluronic acid polymer. The appearance of several new peaks at 2.06, 2.15, 4.51, 4.56, 6.61, 6.67, 6.71, 7.41, 8.13, 8.67, 10.59, and 11.41 ppm corresponded to folic acid structure, in addition to the pluronic acid F127 peaks (mentioned above) confirm the successful synthesis of FA-F127. The unreacted hydroxyl groups appeared at 5.56 ppm (e).

### FT-IR analysis of transferrin conjugated NPs (PMNP-FA-TF)

The FT-IR spectrum of PMNP-FA-TF provided in Fig. 2 (C) shows a broad peak at 3377 cm^−1^ corresponding to the stretching vibration of Fe-OH functional groups on the surface of the magnetic NPs and the peak at 571.5 cm^−1^ is related to the Fe–O bonds of SPIONs.

The characteristic peaks of Pluronic F127 appeared at 1119.6, 1072.3, and 2852.6 cm^−1^, corresponding to C–O–C stretch, CH_2_ vibrations, and methyl groups, respectively^27^. The vibration of the carbonyl group in Pluronic F127 and folic acid conjugation through esterification was detected at 1723 cm^−1^. Various characteristic peaks regarding the structure folic acid were also observed, such as –NH stretching vibration of the pteridine ring, –C=C vibration of phenyl pteridine rings, C=N and amid functional group vibrations, which can be seen at 3148.3, 1456.7, 1600.7, and 1636.1 cm^−1^, respectively^28^. Additionally, the characteristic peaks of transferrin appeared at 1646.9 and 1539.5 cm^−1^, which were attributed to amid I and amid II in its structure, respectively^29^. A shoulder at 1675.6 cm^−1^ further confirmed the successful conjugation of transferrin to the carboxylated-pluronic F127 structure. DLS measurements showed that the average sizes of two types of NPs dispersions in deionized water (DW) were 70.7 and 82.1 nm (without and with VCR, respectively) (Fig. 2D,E). An increase in the size of nanoparticles after drug loading confirmed that vincristine was loaded properly onto the mixed micelles. This was due to the localization of the drug between the hydrophobic domains of the copolymer. The entrapment of drug molecules between hydrophobic layers prevented polymeric chains from crystallization and caused the polymer chains to become farther apart. Relevant studies have reported similar results; for example, Zhang et al. prepared the Paclitaxel-loaded Pluronic P123/F127 mixed polymeric micelles which exhibited a slight increase in size after PTX loading (from 20.1 to 23.5 nm). They explained that very slight mean size increase after PTX loading might nevertheless reflect a certain increase in the hydrophobic micelle core size because of solubilization of PTX^30^.

### SEM and XRD analysis of PMNP-FA-TF nanoparticles

**Figure 3:**
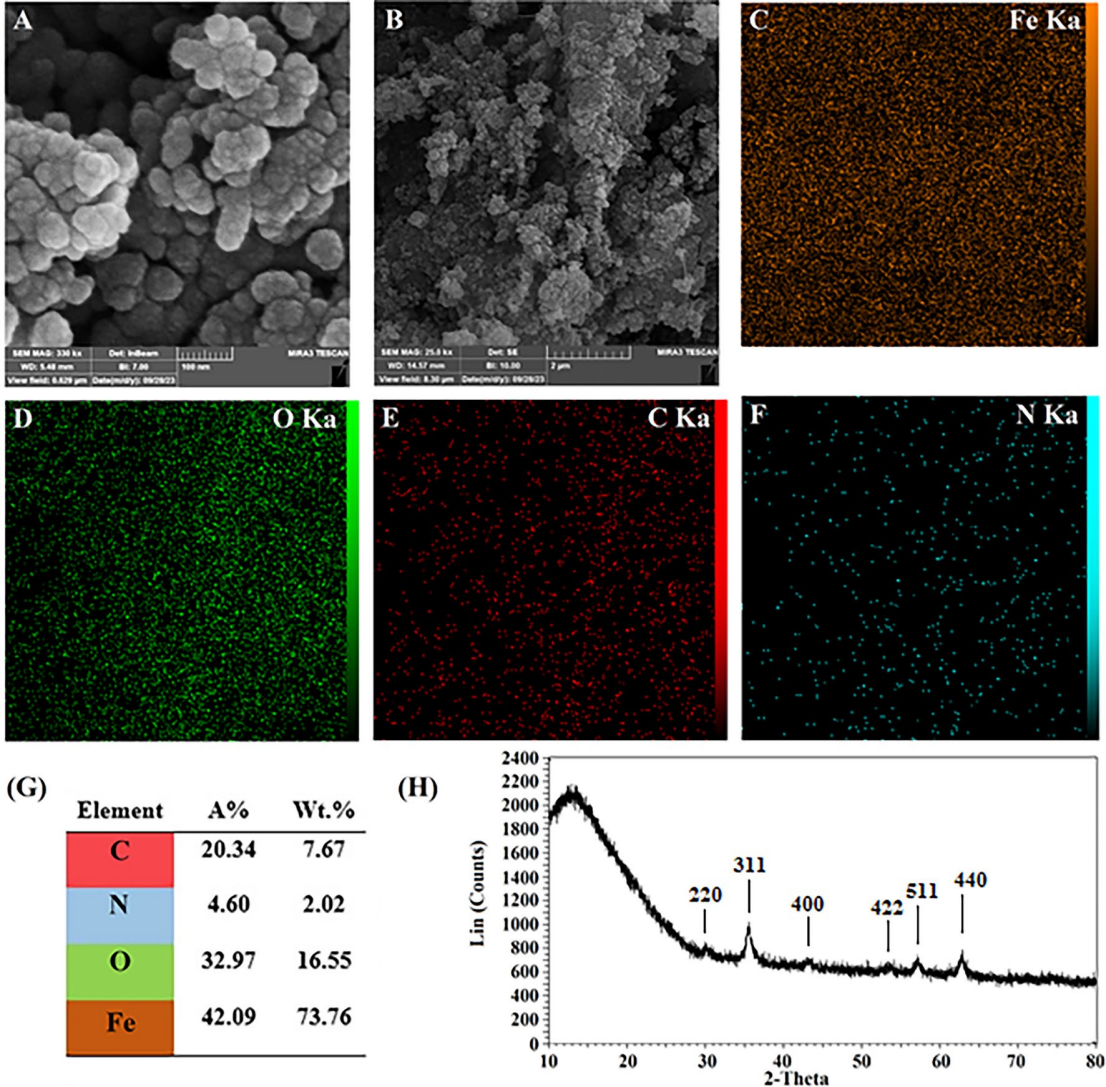
FE-SEM images of final nanoparticles PMNP-FA-TF with two magnifications (**A**,**B**), EDX elemental mapping including Fe, O, C, and N (**C**–**F**, respectively), Elemental composition (**G**), and XRD pattern of final nanoparticles (**H**).

Figure 3A,B present the SEM images of final nanoparticles with two magnifications. According to the zoom-in SEM image (Fig. 3A), PMNP-FA-TF NPs showed a spherical morphology with a size of < 100 nm. The EDX elemental mapping analysis from the zoom-out image (Fig. 3B) exhibited a good distribution of Fe, O, C, and N elements on the synthesized nanoparticles (Fig. 3C–F, respectively). In addition, the elemental composition (in wt.% and atomic percentage (A%)) of the nanoparticles are summarized in Fig. 3G. To confirm the final composition of PMNP-FA-TF NPs, the XRD analysis was carried out (Fig. 3H). The XRD pattern revealed the characteristic peaks of Fe_3_O_4_ nanoparticles, such as (220), (311), (400), (422), (511), and (440) which were assigned in the XRD pattern^31^. The broad peak observed at 2θ = 10°–28° was attributed to the amorphous functionalized Pluronic F127^32^. It was observed that the semi-crystalline structure of Pluronic F127 polymer, which had two diffraction peaks at 19° and 23°, changed into an amorphous structure after conjugation with two ligands (folic acid and transferrin). This structure deformation resulted in the appearance of a broad peak^32^.

### TEM analysis of PMNP-FA-TF nanoparticles

**Figure 4:**
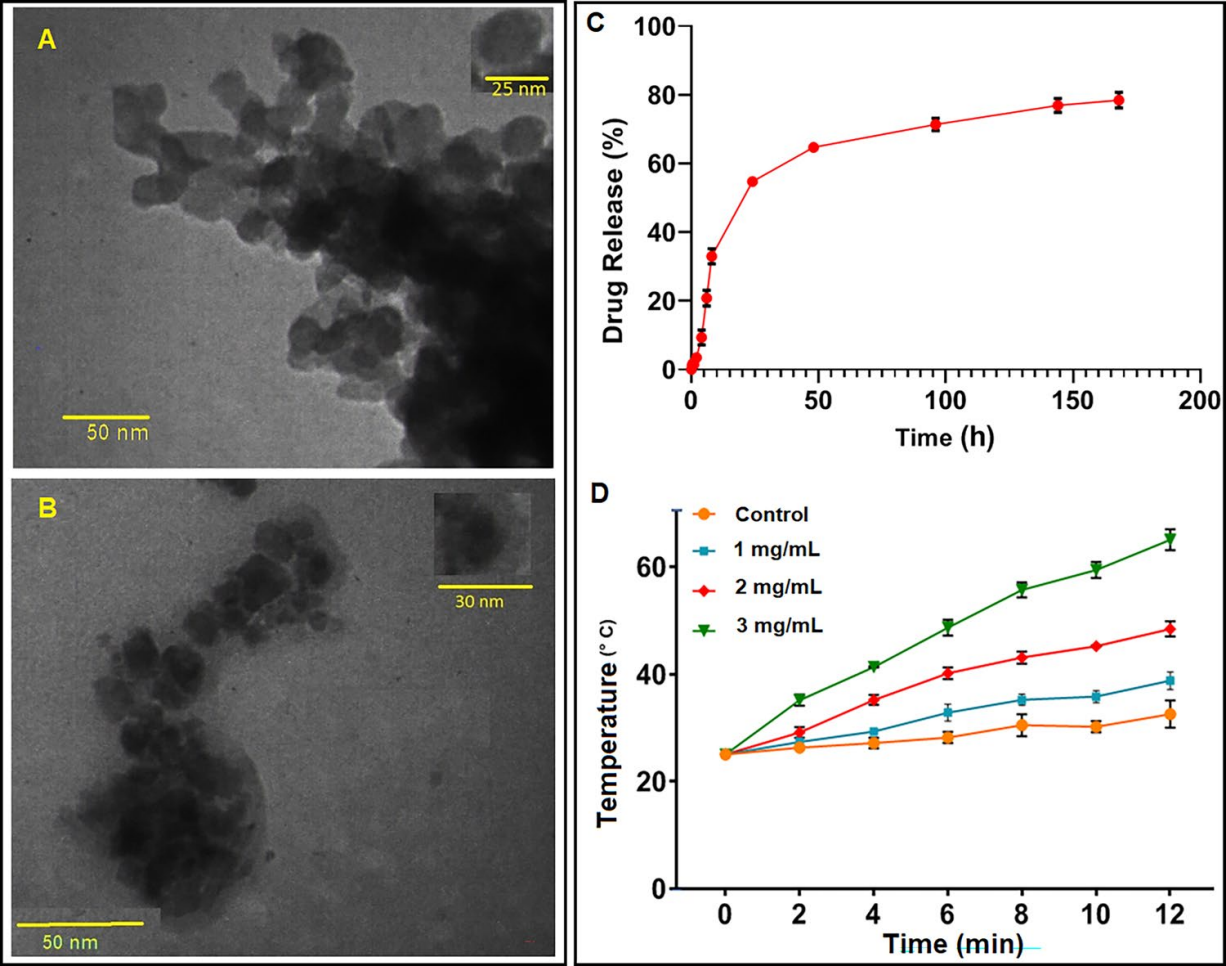
Properties of nanoparticles. TEM images of PMNP-Tf-FA (**A**) and PMNP-Tf-FA-VCR (**B**), In vitro vincristine release profile in 37 °C (**C**), Thermometry of different concentrations of PMNPs and control groups by IR camera during 12 min (**D**).

According to the TEM analysis of PMNP-FA-TF (Fig. 4A) and PMNP-VCR-FA-TF (Fig. 4B), it was found that all the nanoparticles had a spherical core–shell structure and were uniform, with dimensions of approximately 25 and 30 nm. The results from TEM demonstrated lesser value than those obtained from the DLS technique because the latter measures the hydrodynamic size of nanoparticles, while the first method depicts the nanoparticles' size in their dry state. The size, zeta potentials, and other properties of various NPs are summarized in Table 1. The zeta potential of drug-free NPs after transferrin conjugation (PMNP-TF-FA NPs) is + 6.3 because the negatively-charged succinic acid residues might have reacted with positive transferrin agents. This parameter increased to + 25 mV for drug-loaded NPS (PMNP-TF-FA-VCR NPs) due to the insertion of positively charged vincristine into the structure of nanoparticles^33^. In addition, The VCR encapsulation efficiency of the PMNP-VCR-FA-TF nanoparticle was 87.15%, with a drug loading coefficient of 8.7%.

**Table 1:** The properties of nanoparticles.

Properties	PMNP-FA-TF-VCR	PMNP-FA-TF
Hydro dynamic diameter (nm)	82.1	70.7
Zeta potential (mv)	+ 25	+ 6.3
Drug loading capacity (%)	8.7	–
Encapsulation efficiency (%)	87.15	–

### In vitro evaluation of VCR release profile from PMNP

The cumulative in vitro VCR release profile of PMNP-VCR-FA-TF at pH ~ 7.4 and 37 °C is shown in Fig. 4C. After 48 h, the cumulative release rate was 64.71% for PMNP-VCR-FA-TF. We concluded that VCR-loaded NPs exhibited a sustained-release function. This can be described as slow drug release as the polymer dissolves.

### Efficiency of absorption of nanoparticle of heat during AMF

To determine the thermomagnetic performance of PMNPs, suspensions with different nanoparticle concentrations (1, 2, and 3 mg/mL) were exposed to AMF for 12 min and the temperature alteration was checked with an infrared camera. The temperature–time curve at 70 W, while exposed to AMF, is shown in Fig. 4D.

The heating efficiency of PMNP during AMF was determined by calculating the SAR value. The SARs calculated from the estimations were 117.4, 127.5, and 136.1 W/g Fe, respectively (Table 2). SAR values were positively associated with the concentration of PMNPs nanoparticles. These results confirmed that PMNPs might play the role of thermal enhancers in converting magnetic energy into heat. This indicates that magnetization increases with increasing Fe content in nanoparticles, and SAR content also increases with increasing Fe concentration in nanoparticles.

**Table 2:** SAR parameter values calculated based on the SAR formula (Eq. 3).

MNPs concentration	Time (min)	ΔT (°C)	H (A/m)	f (MHz)	SAR (W/g)
1 mg/mL	12	12	40	13.56	117.4
2 mg/mL	12	22.1	40	13.56	127.5
3 mg/mL	12	38.4	40	13.56	136.1

### Hemolysis assay

**Figure 5:**
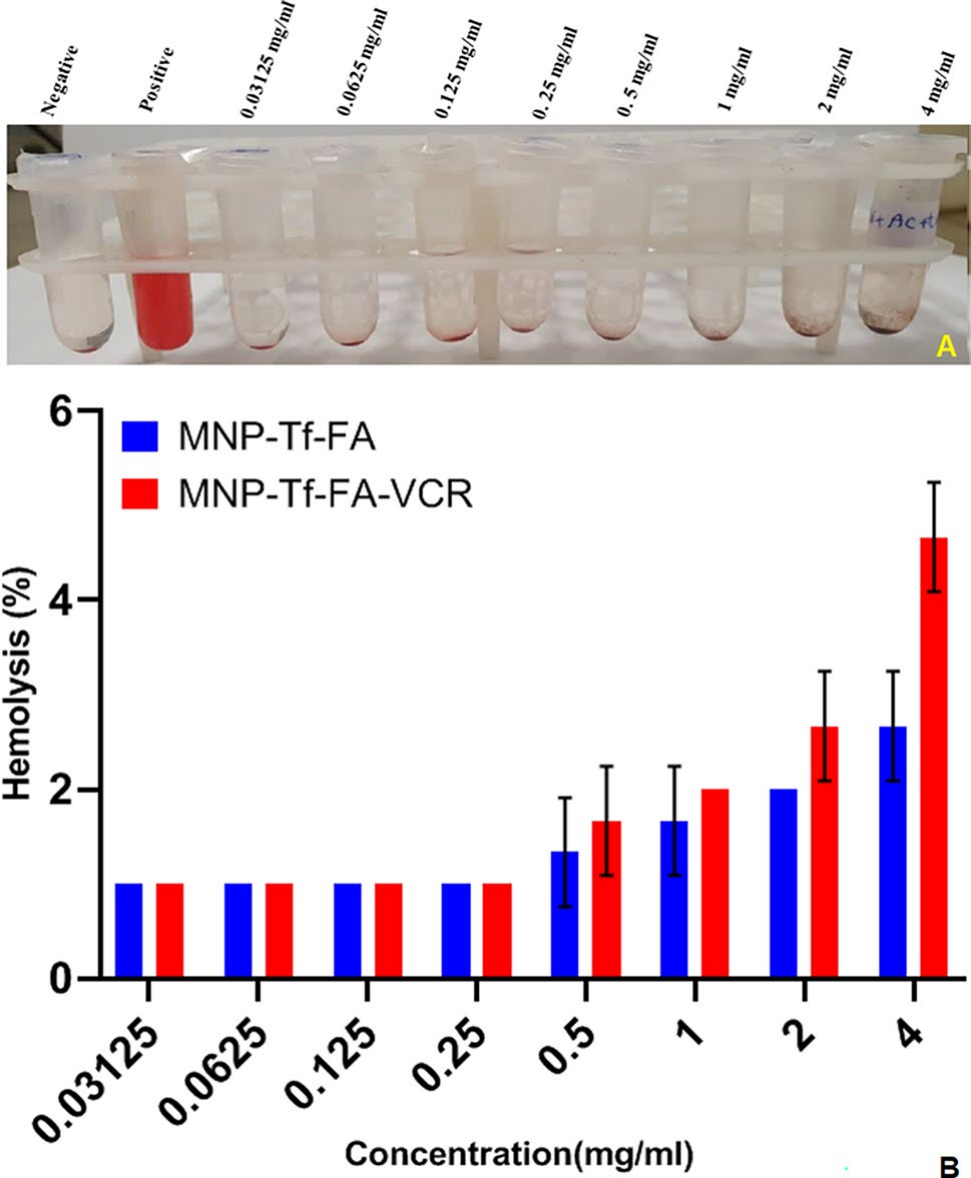
Hemolysis caused by different concentration of nanoparticles. (**A**) Image of samples after centrifugation at 1500 rpm for 5 min: Negative control (PBS), positive control (water), and nanoparticle suspension (4, 2, 1, 0.5, 0.25, 0.125, 0.0625 and 0.3125 mg/mL). (**B**) Evaluation of the percentage of hemolysis of RBCs induced by nanoparticles.

To assess whether the interaction between nanoparticles and red blood cells may cause red blood cell damage, a hemolytic assay was performed. As shown in Fig. 5, the percentage of hemolysis induced was less than 5% at all concentrations used. According to this criterion, a hemolysis rate of less than 5 is considered hemocompatible^34^, meaning that our nanoparticles can be used safely.

### In vitro cellular toxicity

**Figure 6:**
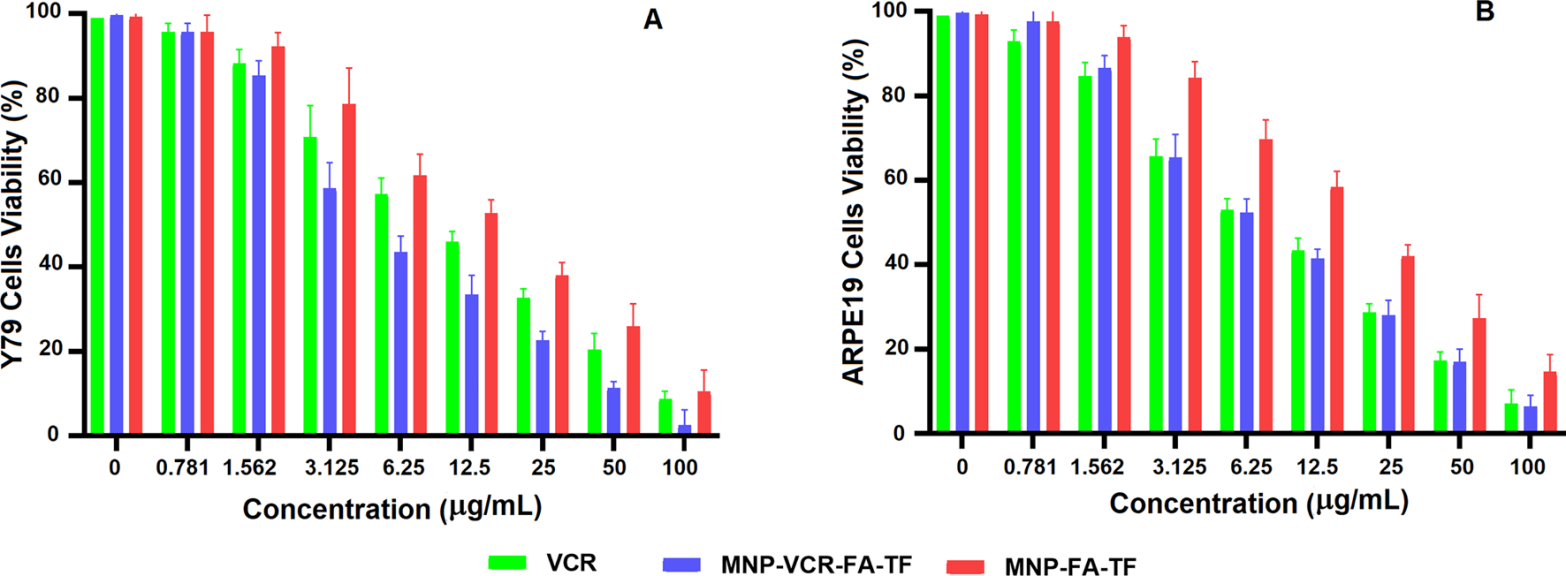
Cytotoxicity of different concentration of VCR, PMNP-VCR-FA-TF and PMNP-FA-TF after 48 h incubation with Y79 (**A**) and ARPE-19 (**B**) cell lines.

Preliminary cytotoxicity of prepared magnetic nanoparticles was assessed with 3-(4,5-dimethylthiazol-2-yl)-2,5-diphenyl tetrazolium bromide (MTT) assay using Y79 cancer cells and normal ARPE19 cells. Moreover, the effect of two types of NPs (PMNP-VCR-FA-TF and PMNP-FA-TF) and vincristine alone on the average proliferation of cancer and normal cells showed a dose-dependent pattern, indicating a decrease in MTT signaling compared with untreated cells. Therefore, within the MTT assay, the Y79 cancer cells and ARPE19 normal cells were incubated with 0–100 μg/mL of nanoparticles and vincristine for 48 h. According to Fig. 6, the results of MTT assay showed that NP without VCR has a significantly lower effect on the viability of cancer and normal cell lines compared to drug and drug-carrying nanoparticles (P > 0.05). As shown in Table 3, the IC50 analysis showed that the normal cells (ARPE19) were more sensitive to the VCR than the cancer cells (Y79). Cell viability was significantly lower after 48 h of incubation with VCR-loaded nanoparticles, indicating that the VCR loaded on nanoparticles was well translocated into the cells (P < 0.05). Especially, the level of toxicity in Y79 cells was significantly higher than ARPE19 cells, which indicated the efficiency of ligand/receptor transfer by folic acid and transferrin. These results suggested that, firstly, PMNP-VCR-FA-TF significantly reduced survival compared to free drugs, which could be attributed to accelerated endocytosis and better drug delivery to cells. Secondly, it caused a decrease in survival in Y79 cells compared to ARPE19 cells that were also infected, this may be due to the presence of transferrin and folate ligands and the presence of numerous receptors for these ligands on the surface of cancer cells.

**Table 3:** IC10 and IC50 of Y79 and ARPE-19 cell lines calculating based on MTT assay.

Cell line	IC10 (µg/mL)		IC50 (µg/mL)	
	VCR	PMNP-VCR-FA-TF	VCR	PMNP-VCR-FA-TF
Y79	1.02	0.98	10.61	4.87
ARPE-19	0.96	1.23	8.91	7.75

### Calculation of heating profile and heat dose with AMF

**Figure 7:**
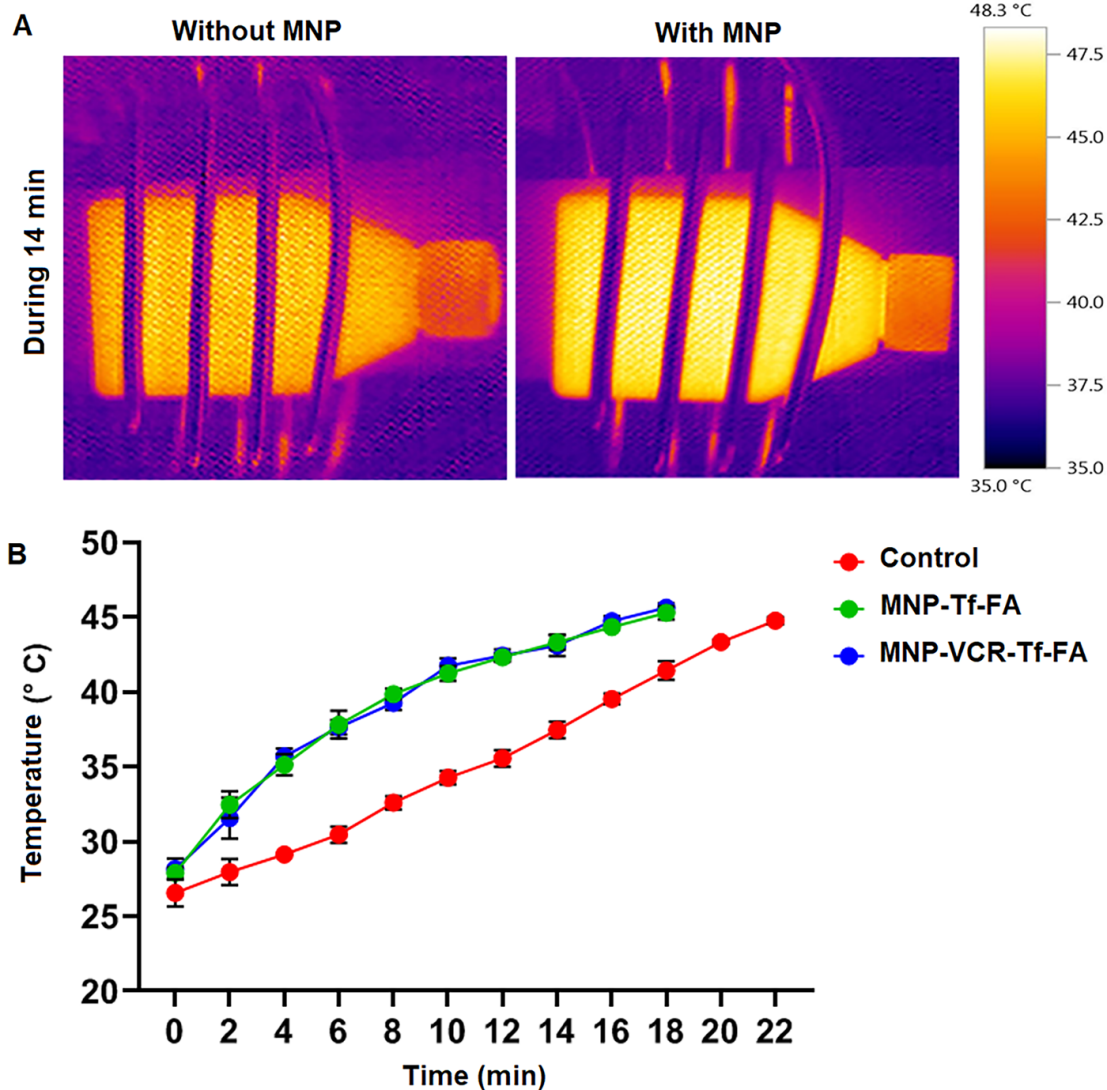
IR camera images of Y79 cells treated with and without nanoparticles under AMF (70 W, 13.56 MHz, 14 min) (**A**), Heating Profile of Y79 cells during AMF exposure with and without PMNP incubation (**B**).

To investigate how long Y79 cells should be exposed to a temperature to reach 43 °C, we examined how the temperature of the cells changed when exposed to AMF, the results of which are shown in Fig. 7. Upon exposure to AMF for 20 min, the temperature of untreated Y79 cells reached 43 °C. The temperature of the nanoparticles used in treatment increased quickly to 43 °C within 14 min (Fig. 7B). For magnetic hyperthermia, the cell flask was put in to a coil after treatment with magnetic nanoparticles (Fig. 7A, 70 W). We used an infrared camera to measure the temperature change in the flask and the temperature–time plot is appearing in Fig. 7B. The presence of NPs (PMNP-VCR-FA-TF and PMNP-FA-TF) resulted in a significant temperature increase compared to the NP-free condition (P < 0.0001). Without nanoparticles, the temperature rise reached a therapeutic temperature of 43 °C for an extended period of time. The diagram showed that cells were placed in AMF for 20 min with AMF treat lonely and 14 min with magnetic hyperthermia treatment (Fig. 7B). This indicates that the presence of NPs in cells significantly increases the temperature under AMF irradiation. We tested the amount of heat to measure the thermal dose retained by Y79 cells with and without MNPs, we considered CEM 43 °C for both at different time focuses, and the results appear in Table 4.

**Table 4:** Thermal dose received by cells from AMF exposure at different time intervals with and without PMNPs.

Treatment	2 min	10 min	Time to 43 °C (min)
AMF	2.21 × 10^−8^	4.38 × 10^−5^	0.1894
AMF + PMNPs	2.86 × 10^−6^	0.9592	0.7578

### Measurement of ROS production by Y79 cells

**Figure 8:**
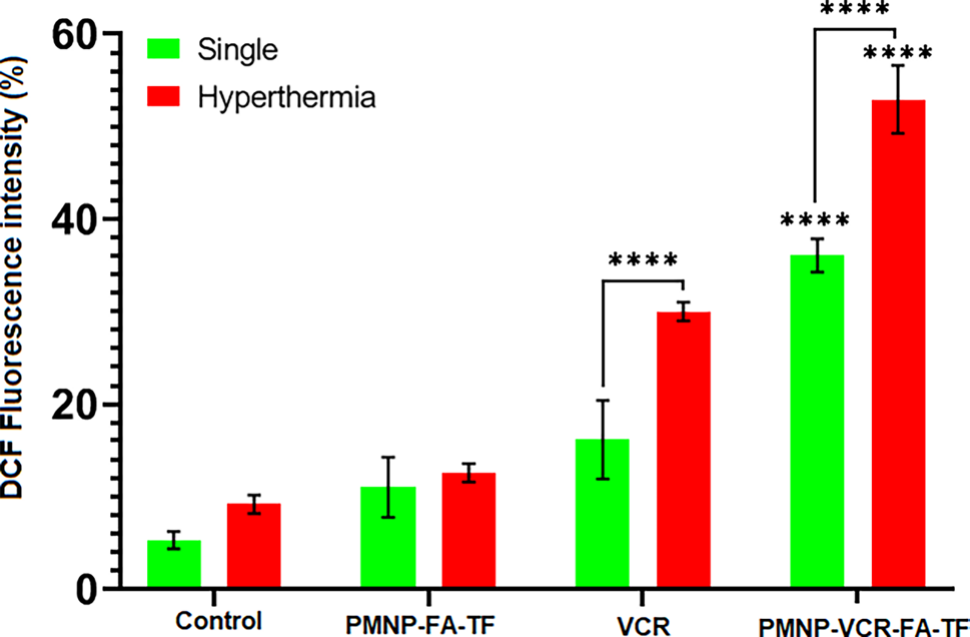
Percentage of DCF fluorescence intensity that represents the intracellular ROS after different treatments (****P < 0.0001).

DCF fluorescence intensity was measured in reaction to different processes to determine the amount of ROS produced in Y79. As shown in Fig. 8, the concentration of intracellular ROS was increased in PMNP-VCR-FA-TF compared to PMNP-FA-TF (P < 0.0001). A significant increase in DCF was moreover observed when PMNP-VCR-FA-TF and VCR was combined with AMF hyperthermia (P < 0.0001). These results confirmed the positive effect of alternating magnetic field application on ROS production in the presence of drugs or nanoparticles. The difference between the two groups of nanoparticles and drug in ROS production with or without AMF indicated the significant effect of nanoparticles on ROS production compared to the drug alone (P < 0.0001). This effect can also be the reason for the toxicity of nanoparticles. This implies that the NPs absorb electromagnetic energy. Finally, the highest amount of ROS (an 8.2-fold increment compared to the control) was observed in the case of PMNP-VCR-FA-TF + hyperthermia.

### Colony formation assay

**Figure 9:**
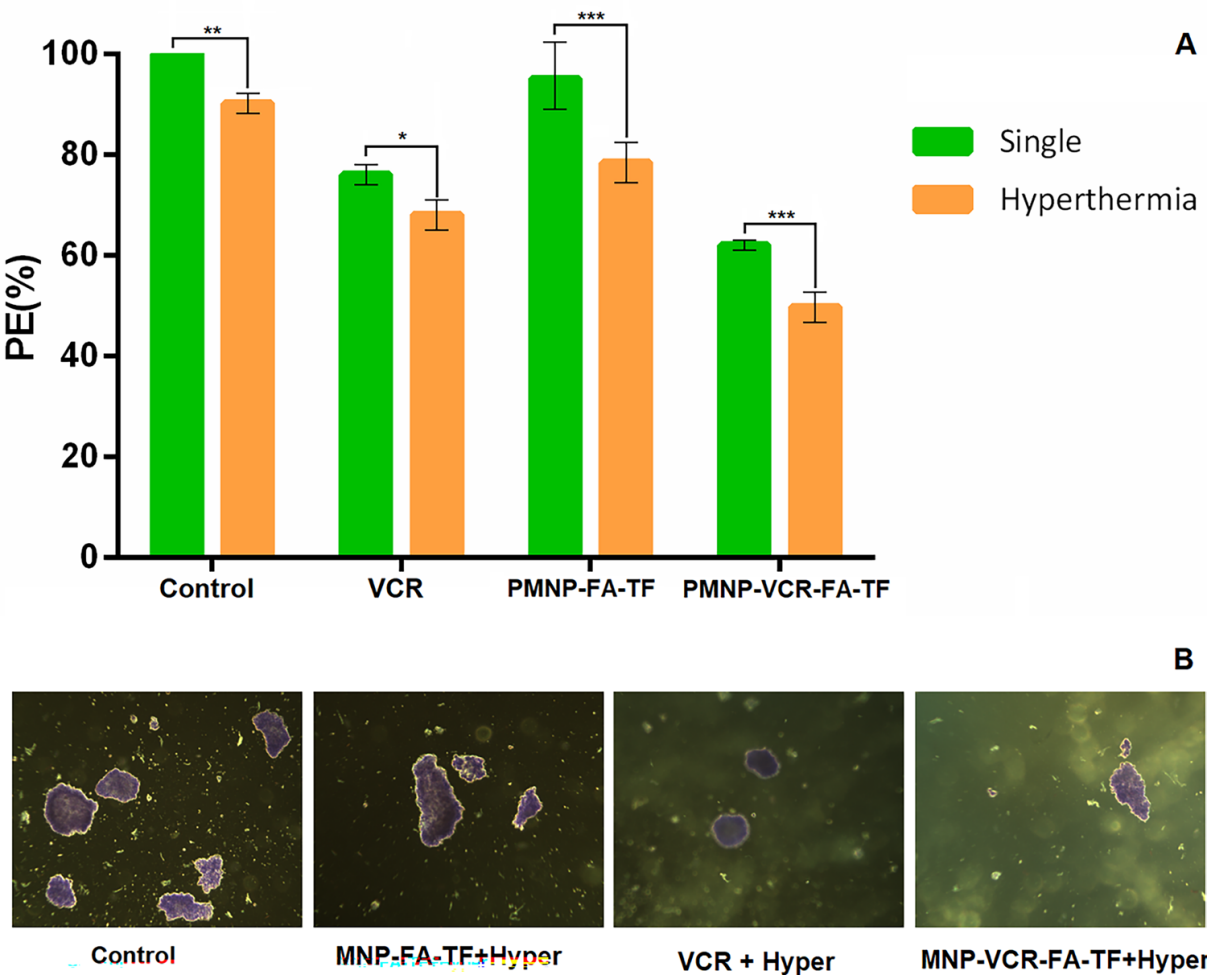
Colony formation assay after treatment whit two types of NPs (PMNP-VCR-FA-TF and PMNP-FA-TF) and vincristine with and without AMF hyperthermia (*P < 0.05, ***P < 0.001, ****P < 0.001) (**A**). Image of Y79 cell colonies under light microscope with 10 × magnification (**B**).

The effect of two types of NPs (PMNP-VCR-FA-TF and PMNP-FA-TF) and vincristine with and without AMF on the Y79 cells colony formation ability is shown in Fig. 9. As shown in Fig. 9, the plating efficiency (PE) of PMNP-FA-TF after 24 treatments was not significantly different from that of control cells, indicating that these NPs have excellent biocompatibility. On the other hand, VCR-carrying PMNP-FA-TF nanoparticles showed a significant decrease in the amount of colonization compared to blank nanoparticles (P < 0.001). This inhibitory effect of colonization under AMF showed a significant increase (P < 0.001). The results showed that the highest rate of inhibition of Y79 cell colonization was in the group of PMNP-VCR-FA-TF under alternating magnetic field, which indicates the potential of combined treatment of hyperthermia with chemotherapy. In addition to assessing the colony formation ability of cells (PE%) in different treatment groups, this factor was subsequently used to obtain treatment ratios. Nanoparticles using drug delivery potential and thermal activation through AMF exposure resulted in a cure ratio of 1.21 for chemotherapy, 1.24 for hyperthermia, and 1.41 for combination chemo- hyperthermia.

## Discussion

According to previous studies, the major challenges in chemotherapeutic treatment of retinoblastoma are the blood-retinal barrier (BRB) and blood–brain barrier (BBB), which limit the entry of drugs at their therapeutic concentrations into the tumor^9^. Over the past decade, nano drug delivery platforms have represented an innovative approach as nano carriers to penetrate the BBB. Various studies have shown that nanoparticles with an average size of less than 100 nm have a high ability to cross the BBB and the BRB^35,36^. Despite the key role of nanoparticle size, target selection and local delivery are critical factors in designing nano drug delivery systems for non-invasive diagnosis and treatment of retinoblastoma^37^.

To overcome these limitations, in this study, we successfully synthesized dual ligand/receptor magnetic nanoparticles in order to improve active targeted drug delivery performance and specific uptake of nanoparticles in cancer cells compared to healthy cells. These polymer nanoparticles (PMNP-VCR-FA-TF) were designed in a way to be capable of delivering a significant dose of VCR chemotherapeutic drug to Y79 retinoblastoma cancer cells based on key advantages including an average size of 80 nm (Table 1) and the use of two ligands, i.e., folic acid and transferrin (Fig. 2). The loading rate of VCR onto nanoparticles was about 9%, which increased the size of nanoparticles from 70.7 to 82.1 nm. An increase in the size of nanoparticles due to drug loading has also been reported by other studies. For example, Minaie et al. showed that TMZ loading on NPs could expand their size from 24 to 44 nm^38^. Since chemotherapeutic drugs have a short half-life in the body, another advantage of loading drugs onto nanoparticles is the ensuing increase in the half-life of drugs in general circulation or tissues secondary to sustained release of the drug. When the drug was loaded onto PMNPs, its half-life increased, as confirmed by the release profile of the drug which encompassed 48 h. The findings of Bahrifar et al. were also a confirmation of this issue, as they reported that the biological half-life of Streptokinase had also increased by twofold in when loaded onto PEG-grafted chitosan^39^.

To release the drug locally, it is necessary to deliver the drug specifically to the tumor area, and to this end, we used PMNP-VCR-FA-TF nanoparticles with two ligands. Transferrin and folic acid are both very effective ligands in targeted medicine and both of them are highly expressed in cancer cells^17^. Previous studies have shown that the expression level of folate and transferrin receptors in Y79 cancer cells is higher than in normal ARPE-19 cells^40,41^. Therefore, PMNPs might be suitable vehicles with excellent performance, efficient cellular uptake and sustained intracellular drug release properties. Our results showed that the IC50s of VCR-loaded NPs (PMNP-VCR-FA-TF) against Y79 were significantly lower than ARPE-19 (P < 0.05, Table 3). This could be due to more drug penetration into Y79 cancer cells than healthy ARPE-19 cells by ligand/receptor targeted delivery^42^. Also, due to the overexpression of folate receptor (in ovarian cancer, colon cancer, epithelioid carcinoma of the cervix and epidermoid carcinoma of the larynx, brain)^43,44^ and transferrin receptor (in breast, bladder, lung adenocarcinoma and chronic lymphocytic leukemia cells)^45,46^ in different types of cancer cells, this nanoplatform could be used for loading different chemotherapeutic agents and deliver drugs to different tumors.

However, even with targeted drug delivery and the improvement in the chemotherapeutic benefit by nanoparticles, chemotherapy alone is not sufficient and should be coupled with an adjuvant treatment. After chemotherapy, cells may become drug-resistant, which can be overcome in part by hyperthermia that renders tumor cells more sensitive by facilitating the S phase of the cell cycle, resulting in more extensive cell death^47,48^. Based on this rationale, we tried to synthesize a distinct set of nanoparticles magnetic hyperthermia that would be capable of heat sensitization. Our results (Tables 2 and 4) confirmed that PMNP-VCR-FA-TF not only could effectively deliver and release VCR, but also induced more heat during hyperthermia by facilitating cell entry, thereby improving effectiveness. According to our thermometric data and SAR calculations, we found that nanoparticle concentration was positively associated with SAR values (Fig. 4D, Table 2). Additionally, our findings also confirmed that the time required for attaining a temperature of 43 °C was shorter in the presence of PMNPs (Fig. 7). Beside heat sensitization, PMNPs are also capable of stimulating Reactive Oxygen Species (ROS) generation especially in the presence of heat, which is thought to be due to the presence of iron oxide VCR. Therefore, to confirm this hypothesis, we evaluated the concentration of ROS after treating Y79 cells with chemotherapeutic nanoparticles using DCFH-DA staining. The findings derived from the treatment of Y79 cells, as depicted in Fig. 8, indicated that the administration of free VCR and hyperthermia separately did not exert a significant effect on the production of ROS compared to the control group (P > 0.001). In terms of differentiation, combined application of VCR and hyperthermia was found to have significant positive regulatory effect on ROS, which was further accentuated by tenfold in cells treated with magnetic hyperthermia and PMNP-VCR-FA-TF, compared with control group (P < 0.001).

Ultimately, in order to investigate the therapeutic efficiency of Y79 retinoblastoma cancer cells under different modalities, the final effect of treatment was assessed with different tests. The results of soft agar colony assay showed that Y79 cells were significantly deprived of their reproductive capacity in the presence of VCR or PMNP-VCR-FA-TF compared to untreated cells (P < 0.05), which can be seen in Fig. 9B. In any case, the impact of PMNP-FA-TF was lower than the VCR alone after 24 h (P < 0.05), which could be due to the superior biocompatibility of PMNPs, as they are generally considered nontoxic or of low toxicity^49^. Consistent with our results, Song et al. found that MSN-FA nanoparticles essentially diminished the number of colony arrangements compared to the control group, while free Myr and Myr-MRP-1/MSN had no significant impacts on the expansion of lung cancer cells^50^. We found that hyperthermia treatment in the presence of drug-carrying nanoparticles led to maximal decrease in the colonization of cells (P < 0.001, Fig. 9A), suggesting a synergistic effect for heat on the effectiveness of the chemotherapeutic drug in question. This finding confirmed the significant role of magnetic hyperthermia as an adjuvant treatment alongside chemotherapy. Various factors may underlie the increased efficacy of hyperthermia and chemotherapy in combination, including the positive regulatory effects of heat on drug release^51^, ROS production (in the presence iron oxide nanoparticles)^52^, and cell death in chemotherapy-resistant cells^53^. Several studies have shown that vincristine stimulates apoptosis by upregulating ROS generation^54,55^. In this regard, our findings were in good agreement with colony assay, showing that AMF + VCR-PMNP-FA had resulted in the highest rate of cell death due to improved targeting (P < 0.0001).

## Conclusion

In this study, we successfully developed a magnetic nanocarrier of Vincristine based on the active targeting system of drug delivery using dual folic acid/transferrin ligand-containing PMNPs, namely, PMNP-VCR-FA-TF, the efficacy of which was tested on Y79 retinoblastoma cells. The results of nanoparticle characterization confirmed that these nanoparticles had suitable size, low toxicity and good biocompatibility, while being capable of targeting Y79 cancer cells compared to ARPE-19 normal cells. After being exposed to an AMF, the nanoparticles effectively generated localized heat and reduced the colony formation ability of cancer cells through the release of the VCR, while significantly enhancing the chemotherapeutic efficiency due to the change in the magnetic moment of nanoparticles. As such, the present study delivers evidence that magnetic hyperthermia can be therapeutic value as an adjuvant treatment alongside chemotherapy.

## Methods and materials

### Material

RPMI cell culture medium, Fetal Bovine Serum (FBS), and Penicillin–streptomycin, Trypsin and EDTA were purchased from Biowest (France). Vincristine (VCR), Folic acid, N,N′-Dicyclohexylcarbodiimide (DCC), 4-dimethylamino pyridine (DMAP), Dimethyl sulfoxide (DMSO), 1-Ethyl-3-(3-dimethyl aminopropyl) carbodiimide (EDC), N-Hydroxy succinimide (NHS) 98%, iron (II) chloride tetrahydrate (FeCl_2_(5H_2_O)) and iron (III) chloride hexahydrate (FeCl_3_(6H_2_O)) were purchased from Sigma (Sigma Chemical Company (St. Louis, MO, USA).

### Methods

#### Nanoparticle synthesis

##### Synthesis of superparamagnetic iron oxide NPs

Chemical coprecipitation method was used for the synthesis of superparamagnetic iron oxide nanoparticles^56^. At first, FeCl_2_·5H_2_O (1 g, 0.0046 mol) and FeCl_3_·6H_2_O (2.6 g, 0.0096 mol) were dissolved in deionized water in a flask with a nitrogen gas inlet and a mechanical stirrer. Once the reaction temperature reached 70 °C, ammonia (10 mL) was gradually and slowly introduced into the reaction mixture by dripping. After the ammonia addition was finished, the reaction was sustained for 1.5 h under a nitrogen atmosphere and stirring at a speed of 420 rpm. The obtained product was washed three times with water (3 × 20 mL) and twice with ethanol (2 × 20 mL) to remove impurities. The obtained nanoparticles were dried in a vacuum oven at a temperature of 45 °C for 48 h, and the product was obtained with a yield of 96%.

##### Preparation of oleic acid coated Fe_3_O_4_ nanoparticles

1.2 g of iron oxide nanoparticles were dispersed in 35 mL of deionized water using ultrasonic waves for 5 min. About 0.5 mL of oleic acid was added to the above dispersion and sonicated for 3 min. Then, 6 mL of ammonia (25 wt%) was added, and homogenization was continued for 5 min. The homogenized solution was stirred with a mechanical stirrer for 120 min. After the indicated time, the excess ammonia was neutralized with HCL. Finally, the oleic acid coated nanoparticles were separated using a centrifuge at 3000 rpm. The obtained nanoparticles were washed once with ethanol–water solution (in a ratio of 1:3) and twice with ethanol–water solutions (in a 1:1 ratio) to remove impurities, and dried in a vacuum oven at a temperature of 50 °C for 48 h (yield = 87%).

##### Synthesis of folate-pluronic F127 (FA-F127)

Folic acid was activated using DCC (4.5 mg) and DMAP (1 mg) in 10 mL DMSO, under dark conditions and at room temperature for 24 h. Then 63 mg of dried pluronic F127 was added to the reaction mixture and homogenized by magnetic stirring for another 24 h under the same conditions. The final product was obtained by centrifuging the mixture at 3000 rpm for 5 min and then drying by a freeze dryer. The crude yellow powder was dialyzed in distilled water using a 12 kDa dialysis bag for 3 days to remove the free folic acid and other small molecules impurities, and then lyophilized to produce folate conjugated F127 with 85% yield.

##### Synthesis of carboxylated-pluronic F127 (COOH-F127)

63 mg (5 μmol) of freshly dried pluronic F127 in 15 mL of dried toluene was activated by 1 mg (8 μmol) DMAP at 70 °C under nitrogen atmosphere. The reaction mixture was stirred for 30 min after the addition of two drops of triethylamine as a catalyst. Subsequently, 12.5 mg (0.12 mmol) of succinic anhydride was introduced directly into the reaction mixture. Following 24 h, the mixture was precipitated in cold diethyl ether, separated through filtration, pure ether washed, and air-dried to yield COOH-F127 with a 91% yield.

#### Preparation of transferrin-decorated mixed micelles (PMNP-FA-TF) with and without vincristine

##### Preparation of hydrophobic vincristine drug

Until the pH of the drug solution reaches 7, Triethylamine was added dropwise to the Vincristine sulfate solution in water (1 mg/mL). Then the hydrophobic drug precipitated in the aqueous solution was separated and then dried through lyophilization under reduced pressure.

##### Preparation of COOH-F127 and FA-F127 mixed micelles with SPIONs core (PMNPs)

A mixture of 96 mg of carboxylated-pluronic and 96 mg of folate-pluronic in 4 mL of dry THF was added dropwise to a dispersion of 40 mg of oleic acid-coated magnetic iron oxide nanoparticles in 100 mL of distilled water (using an ultrasonic probe with 5 s on and one second off, for 5 min and 40% power) at ambient temperature. After 15 min of stirring, the organic solvent was removed using a rotary evaporator, and the magnet was used to collect the functionalized polymer-coated iron oxide nanoparticles, which were then washed with water and dried at room temperature.

##### Preparation of drug-loaded mixed micelles with SPIONs core (PMNPs)

In this section, 96 mg of carboxylated pluronic and the same amount of folic acid terminated pluronic were dissolved in 4 mL of dry THF containing 4 mg of hydrophobic vincristine. The prepared mixture was added dropwise to a dispersion of 40 mg oleic acid-coated magnetic iron oxide nanoparticles in 100 mL of distilled water. The process continued in the same way as the previous part.

##### Preparation of transferrin conjugated nanoparticles without drug (PMNP-FA-TF)

For this purpose, 40 mg of the prepared mixed micelles containing SPIONs from previous step were dispersed in 10 mL of distilled water using an ultrasonic probe for 2 min and 40% power. Then, the carboxyl groups of Pluronic F127 on the surface of NPs was activated by adding 2.1 mg of NHS and 4.5 mg EDC and stirring for 2 h at room temperature for the amidation reaction with Transferrin. Then, 1 mg of transferrin was dissolved in 1 mL of cold phosphate buffer and added to the reaction mixture. After 24 h, the final nanoparticles were collected by a magnet, washed with distilled water, and dried by freeze dryer.

##### Preparation of transferrin conjugated nanoparticles containing drug (PMNP-VCR-FA-TF)

This part was done using drug-containing nanoparticles instead of drug-free ones, similar to the previous part.

### Characterization

A perform proton nuclear magnetic resonance (1H-NMR) spectroscopy (Varian Inova, 500 MHz) was performed to evaluate the structure of the synthesized polymers, i.e. carboxylated and folate Pluronic F-127. The samples were dissolved in CDCl_3_ and subsequently subjected to spectral analysis. Final NPs were characterized by a Nicolet 800 Fourier transform-infrared (FT-IR) spectrometer. The morphology of final NPs was analyzed using scanning electron microscopy (SEM, FESEM-TESCAN MIRA3). Energy dispersive X-ray spectroscopy (EDX, FESEM-TESCAN MIRA3) was also used to recognize the elemental composition of final nanoparticles. The X-ray diffraction (XRD) pattern of final nanoparticles were obtained using X-ray diffractometer (XRD Europe 600, GNR, Cu Kα radiation, recorded from 10 to 80). The present study aimed to examine the size distribution and zeta potential of two distinct forms of mixed micelles, namely PMNP-VCR-FA-TF and PMNP-FA-TF, through the utilization of analytical techniques including DLS and zeta potential/particle sizers (Nanoflex, Particle Metrix, Germany). The specimens were subjected to dilution with deionized water at the appropriate concentrations, followed by sonication for a duration of 5 min prior to quantification. The morphology of synthesized nanoparticles was assessed by means of transmission electron microscopy (TEM) employing the Zeiss LEO906 instrument, which is located in Jena, Germany. For this test, nanoparticles were uniformly dispersed in deionized water and subjected to sonication for a duration of 5 min. A droplet of the dispersed sample was applied onto a copper grid covered with a layer of carbon. Subsequently, the grid was subjected to observation via transmission electron microscopy (TEM).

### Vincristine encapsulation efficiency and loading capacity

To obtain the vincristine loading capacity (VLC), and vincristine encapsulation efficiency (VEE), nanoparticles composed of (PMNP-VCR-FA-TF) at a mass of 5 mg were solubilized in a DMSO solution. The quantification of the (PMNP-VCR-FA-TF) nanoparticle's encapsulation efficacy was conducted by means of a Hitachi U-2001 UV–Vis spectrophotometer which enabled the monitoring of the vincristine feature absorption peak at 210 nm. Absorbance values were converted to appropriate concentrations using a vincristine calibration curve. The blank sample was prepared through dissolving the similar amount (5 mg) of (PMNP-FA-TF) nanoparticles in DMSO as solvent. The absorbance values were converted to corresponding concentrations concurring with the calibration curve. The VLC and VEE were determined using the following equations:

1 $$VLC\, (\%)=\frac{weight\, of\, drug\, in\, nanoparticle }{nanoparticle\, weight }\times 100$$

2 $$VEE\, (\%)=\frac{weight\, of\, drug\, in\, nanoparticle }{weight\, of\, drug\, in\, the\, initial\, loading }\times 100$$

### In vitro evaluation of VCR release profile

The release behavior of vincristine was assessed in vitro from the PMNP-VCR-FA-TF formulation using the equilibrium dialysis bag diffusion technique. In this study, the suspension of nanoparticles (6 mg) was subjected to exchange into dialysis bags (MWCO 12,400 Da) after being suspended in phosphate-buffered saline (PBS, pH of PBS ~ 7.4). The experimental procedure involved submerging the dialysis bags in 10 mL of release medium, specifically PBS, and subsequently incubating the system under controlled conditions at a temperature of 37 °C and a shaking speed of 100 revolutions per minute. At predetermined intervals, the medium underwent a process of complete removal, and a commensurate volume of fresh medium was introduced in its place. The vincristine concentrations that were discharged were assessed using a UV spectrophotometer at 210 nm.

### Heat absorption efficiency of nanoparticles during AMF

The absorption efficiency of the material heated in the target tissue by AMF is calculated as the specific absorption rate (SAR). SAR is used to characterize magnetic energy conversion to heat^34^. Increasing the temperature is beneficial for most applications, and magnetic nanoparticles offer high efficiency and suitable properties for converting magnetic energy into heat^57^. Various concentrations (0–3 mg/mL) of PMNP-VCR-FA-TF arrangement were transferred to Petri dishes. A temperature–time curve (ΔT/Δt) was gotten by setting Petri within the center of a copper induction coil and applying AMF at the 70 W power until the temperature come to 43 °C. Temperature changes amid warming were observed with an IR camera. To assess the heating effectiveness of PMNP-VCR-FA-TF (as an attractive nanoheater), the specific absorption rate (SAR, W/g Fe) was calculated according to Eq. (3).

3 $$SAR=\frac{C}{{m}_{Fe}}\times \frac{\Delta T}{\Delta t}$$

The given equation involves the initial slope of the temperature curve that varies with time and is denoted by ΔT/Δt. The specific heat of water, represented as C_Water_ (equal to 4.18 J/g × ℃), and the mass of iron nanoparticles contained in a suspension, m, also form part of the equation.

### Biosafety test

#### Hemolytic toxicity

Blood compatibility was evaluated with a hemolysis assay. Fresh blood was extracted from humans and stabilized with Heparin. Whole blood sample (3 mL) was centrifuged for 5 min at 1500 rpm to settle red blood cells (RBCs). After centrifugation, the plasma was removed slowly with a sampler, PBS (pH ~ 7.4) was added to the remaining sediment and the tube was turned upside down several times. The RBCs were further washed four times with PBS until the surface becomes colorless and then a mixture of blood (1 mL) and PBS (49 mL) is poured into a Falcon tube and moved upside down. After that, 500 µL of the solution (blood + PBS) with 500 µL of nanoparticle suspension in PBS with the following concentrations was transferred into different microtubes. 500 µL of diluted RBC suspension was exposed to 500 µL of the nanoparticle suspension to make the final nanoparticle concentration 4,2,1, 0.5, 0.25, 0.125, 0.0625, 0.03125 mg/mL. Following a 2-h incubation period at 37 °C and centrifugation at 1500 rpm for 5 min, 100 μl of each sample supernatant was transferred to a 96-well plate for subsequent absorbance measurement utilizing a UV–visible spectrophotometer (Biotech, USA). A negative control was established by utilizing a PBS sample at a wavelength of 540 nm, while a positive control was established by utilizing water. The degree of hemolysis was denoted by the hemolysis rate, which was represented by the mathematical Eq. (4).

4 $$Hemolysis\, (\%)=\left(\frac{sample-negative\, control}{positive\, control-negative\, control}\right)$$

### Cell experiments

#### Ethics

For in vitro studies, Y79 and ARPE-19 cell lines were obtained from the Pasteur Institute Cell Bank located in Tehran, Iran, and were cultured under standard conditions. All experiments were performed according to the instructions of the Ethical Committee of Iran University of Medical Sciences (No. IR.IUMS.FMD.REC.1400.076).

#### Cell culture

The Y79 cell line of retinoblastoma and the ARPE-19 cell line of retinal pigment epithelium were cultured under specific conditions. The Y79 cell line was cultured using RPMI medium, while the ARPE-19 cell line was cultured using DMEM medium. Both mediums were supplemented with 10% FBS, as well as penicillin/streptomycin (100 mg/mL). The cultures were maintained at a temperature of 37 °C in a humidified incubator with an atmosphere consisting of 5% carbon dioxide.

#### Evaluating invitro cytotoxicity effect of nanoparticles using MTT

In the present study, we adopted the MTT colorimetric test to evaluate cytotoxicity. In this method, the yellow tetrazolium salt is converted into purple formazan crystals by mitochondrial enzymes in living cells^40^. To determine the cytotoxicity of two nanoparticles, as well as vincristine administered in isolation, we used the MTT assay, which involved seeding of Y79 and ARPE-19 cells at a cellular density of 10^4^ cells per well in 96-well plates, followed by incubation for a period of 24 h. Subsequently, we subjected our cellular specimens to varying concentrations of vincristine in combination with equivalent dosages of empty and substance-incorporated nanoparticles, ranging from 0.6 to 80 micromoles, over a period of 48 h. In the case of adherent cells, the culture medium was removed by aspiration. To process suspended cells, we subjected 96-well plate to centrifugation at a force of 1000×*g* and maintained a temperature of 4 °C, utilizing a microplate-compatible centrifuge for a duration of 5 min, followed by the careful aspiration of the media. Subsequently, the cells were subjected to a treatment regimen involving the application of 5 mg/mL of MTT, followed by the addition of 100 μL of solvent for MTT to each individual well. This incubation took place in a dark environment at a temperature of 37 °C for four hours. In order to process cells in suspension, it is recommended to subject the 96-well plate to centrifugation in a centrifuge compatible with the plate at 1000×*g* and 4 °C for a duration of 5 min. The medium should then be carefully aspirated followed by complete aspiration of the MTT solvent. Subsequently, a volume of 200 µL of dimethyl sulfoxide (DMSO) solvent was introduced into each well of the receptacle. The aforementioned dish was subsequently enveloped with aluminum foil and subjected to 15 min of agitation utilizing an orbital shaker. The measurement of readout absorbance at a wavelength of 590 nm was conducted through the utilization of a microplate reader (BioTek, Winooski, VT). The quantification of the absorbance that is proportionate to cell number has been ascertained by deducting the absorbance detected in the control or background sample from the obtained experimental results. Subsequently, the aforementioned entities were employed to determine the concentration of both vincristine and vincristine-loaded nanoparticles necessary to attain 50% inhibition of Y79 and ARPE-19 cells, denoted as IC50. Cytotoxicity was assessed as a percentage and was determined via the utilization of Eq. (5) by using the corrected absorbance.

5 $$\text{Cytotoxicity }(\%)=\left(\left(\frac{Control}{Sample}\right)-1\right)\times 100.$$

#### Investigation of the heating profile and thermal dose

In order to attain a mean sample temperature of 43 °C, a time–temperature profile was established prior to hyperthermia treatment to determine the requisite duration of AMF exposure. It was deemed necessary to culture Y79 cells in T-25 flasks. Following a 24-h incubation period, the cells were subjected to nanoparticle treatment for a period of 48 h. After that, the nanoparticles that are unincorporated are removed from the flask and replaced with new medium for suspension cells, nanoparticles were collected by using a magnet, and adherent cells by washing with PBS (pH ~ 7.4). The experiment involved positioning the sample at the core of the coil, followed by subjecting it to an AMF (13.56 MHz, 70 W). The temperature variances were documented through the employment of an infrared thermal imaging device. The mean temperatures pertaining to a specific time period were obtained through the identification of the area of interest in the thermal image of the experiment. The mean temperature obtained was subsequently utilized to construct a time–temperature profile. At this juncture, the time–temperature profile was employed to estimate the thermal properties acquired. The cumulative equivalent minutes at 43 °C (CEM 43 °C) is the foremost common thermal measurements estimation parameter speaking to distinctive time–temperature designs of hyperthermia in terms of identical divisions at 43 °C and gives an apparatus for comparing diverse hyperthermia. The CEM temperature of 43 °C was originally proposed by Dewey, utilizing the Arrhenius diagram as a fundamental basis for comparison with Eq. (6).

6 $$CEM\, 43\,^{\circ}C=\sum\limits_{i=1}^{i}t (i){R}^{43-T (i)}$$

The present equation characterizes the sample time interval as t(i) and the mean temperature during a given warming period of i minutes as T(i). The parameter denoted as R exhibits a value of 0.25 at temperatures that are below 43 °C and assumes a value of 0.5 for temperatures that exceed 43 °C.

#### Investigation of anticancer effects of single and combined treatments with vincristine, PMNPs and hyperthermia

The anticancer effects of vincristine, nanoparticles, AMF hyperthermia monotherapy and combination therapy were studied in Y79 cancer cells. The eight groups were therefore treated as follows: (1) Control (No treatment), (2) Vincristine drug at concentration (1.02 μg/mL), (3) Magnetic nanoparticle with ligand (folic acid + transferrin), (4) Magnetic nanoparticle with ligand (folic acid + transferrin) carrying vincristine drug (20.11 μg/mL containing 1.13 μg/mL), (5) Magnetic hyperthermia (70 W, 10 min), (6) Magnetic hyperthermia + Drug, (7) Magnetic hyperthermia + nanoparticles, (8) Magnetic hyperthermia + nanoparticle carrying vincristine drug.

To summarize, Y79 cells were initially seeded at a density of 2 × 10^5^ cells per square centimeter and given various treatments, including vincristine and blank or drug-loaded nanoparticles, after 24 h. Following this, an AMF with a frequency of 13.56 MHz and a power of 70 W was applied for 48 h to the group of cells undergoing hyperthermia, leading to an average temperature of 43 °C. Exposure times were gotten based on temperature measurements. Eventually, we examined the inductive effect by measurement of ROS generation and colony formation assays.

#### Measurement of ROS production

Reactive oxygen species (ROS) are chemically reactive entities that comprise oxygen molecules. The measure of reactive oxygen species (ROS) can be represented by the level of ROS activity within cells through the utilization of a cell-permeable fluorogenic test known as 2′,7′-dichlorodihydrofluorescein diacetate (DCF-DA), a cell-based measure delineated for this purpose. Upon cellular uptake, the DCF-DA molecule undergoes deacetylation by endogenous esterases, resulting in the formation of a non-fluorescent compound. Subsequently, reactive oxygen species (ROS) promote the swift oxidation of the non-fluorescent molecule to produce the fluorescent compound DCF. This process leads to the visualization and measurement of intracellular reactive oxygen species. “The compound DCF exhibits highly fluorescent properties and is capable of detection through the employment of a flow cytometer, utilizing an excitation wavelength of 485 nm and an emission wavelength of 535 nm.” The concentration of fluorescence obtained is proportionate to the concentration of intracellular ROS. In this study, cellular specimens were distributed into quadruplicate wells of four-well plates and subjected to the pre-determined treatment protocol as previously outlined. Upon initiation of treatment, the cell culture was replaced with He-DCFH-DA and subjected to a 20-min incubation period at 37 °C in the absence of light. Subsequent to this, the cells were subjected to two washes utilizing phosphate-buffered saline (PBS, pH ~ 7.4), and then assessed using a flow cytometer.

#### Soft agar colony formation assay

The enduring cytotoxic effects of unique treatment modalities were evaluated through the utilization of the soft agar colony formation assay. Y79 cells were cultivated as individual cellular units within agarose, with the intention of assessing their capacity to form colonies. In summary, a layer of 0.6% agarose, supplemented with RPMI medium and 40% FBS, was introduced into the wells of a 24-well plate. The Y79 cells were subjected to treatment in accordance with the previously reported methodology. Following the application of the treatment, the cellular matter was extracted and subsequently incubated within suspension in wells while being immobilized within a 0.3% agarose medium. The number of cells per treatment was predetermined and precise. The plates were subjected to incubation for a duration of two weeks. The colonies obtained were subjected to fixation using 3.7% paraformaldehyde and subsequent staining with crystal violet. The colonies were subjected to photographic documentation and subsequent analysis. For the purpose of plating efficiency (PE) analysis, colonies containing more than fifty cells were enumerated and the corresponding colony percentages were computed according to the following formula.

7 $$PE\, (\%)=\frac{ \text{Number of the colonies }}{\text{Number of cultured cells}}\times 100$$

These values were further utilized to obtain the therapeutic ratio combination therapy ratio to single therapy (Eq. 8):

8 $$Theraputic\, ratio=\frac{PE\,  of\,  single\,  treatment\,  group }{PE\,  of\,  combined\,  treatment\,  group}$$

### Statistical analysis

The obtained data were subjected to a one-way analysis of variance (ANOVA) and a subsequent Tukey’s post hoc test was conducted for further analysis. The level of significance is established at P < 0.05. The statistical analyses for this study were conducted using GraphPad Software. The outcomes are represented in terms denoted as mean ± SEM.

### Ethics approval and consent to participate

This research was approved by Ethics Committee of Iran University of Medical Sciences. To take a blood sample for hemolysis test, written informed consent to participate in the experiment was obtained from each participant prior to inclusion. All research involving human research participants has been conducted in accordance with the Declaration of Helsinki and the protocols of Iran University of Medical Sciences.

## Data Availability

The datasets used and/or analyzed during the current study are available from the corresponding author on reasonable request.
